# Professionalism in small group learning between face-to-face and virtual settings: a mixed-methods study

**DOI:** 10.5116/ijme.6413.4ecd

**Published:** 2023-03-31

**Authors:** Xiaomei Song, Michael Elftman

**Affiliations:** 1 Center for Medical Education, School of Medicine, Case Western Reserve University, USA; 2 Immunology & Infectious Disease, College of Medicine, Central Michigan University, USA

**Keywords:** Professionalism, case-based learning, team-based learning, virtual, professional identity formation

## Abstract

**Objectives:**

To explore whether and how preclinical medical students changed perceptions and behaviors related to professionalism in small group learning activities from face-to-face to virtual during the pandemic.

**Methods:**

The study used a mixed-methods sequential research design. We first retrospectively examined quantitative data from 101 medical students who completed mandatory peer evaluation surveys assessing professional behaviors of small group members in two courses (one face-to-face, the other online). Differences between student perceptions in two settings were compared using the Wilcoxon signed-rank test. Findings from the quantitative stage were probed further using focus groups at the qualitative stage. Six focus groups (n = 27) were conducted using purposeful sampling. Interviews were transcribed and inductive thematic coding was used to identify emerging themes.

**Results:**

We found a significant decrease in perceptions of punctuality and attendance in the virtual setting compared to face-to-face learning (Z=-6.211, p<.001), despite lower expectations of their peers in online learning. Five major themes emerged from the qualitative data: punctuality/participation, camera usage, dress code/conversational style, multitasking, and engagement/accountability. Participants showed sensitivity when conceptualizing professional conduct, indicating the dynamic process of professional identity formation at the early stage of their career.

**Conclusions:**

Results show that students’ perceptions of professionalism become contextualized, significantly influenced by the background of the virtual learning environment. Intentional communication about professionalism within specific sociocultural and educational contexts is vital for individual professional identity formation. These findings support of the importance of considering context when educational programs develop curricula and establish expectations related to professionalism.

## Introduction

Professionalism is one of the core competency domains of medical practice around the world. Over the last few decades, medical professionals and organizations proposed various frameworks of professionalism, and medical schools have designed and developed various curricula, instructional approaches, and assessment methods to address this core competence with medical students.^1^^, ^^2^ There is a consensus about the importance of professionalism in medical education; however, no agreement has been achieved about essential knowledge and skills required to demonstrate professional conduct as well as valid assessment tools to assess professionalism.^3^

The COVID-19 pandemic further complicated the situation when many medical schools shifted from face-to-face to online settings. While most literature on medical student professionalism focuses on the attitudes and behaviors of students in either the classroom or clinical setting, little is known about students’ perceptions and behaviors related to professionalism in the online setting. It is not clear whether students demonstrate professional conduct differently in an online setting due to the lack of a physical presence. Using a mixed-methods sequential design, we compared professionalism in two settings based on peer evaluation and focus group results and investigated whether and how students changed their perceptions and behaviors because of the online setting.

### Professionalism in small groups

Professionalism has been defined in a variety of ways. For example, the Physician Competency Reference Set provides a list of common learner expectations utilized in the training of physicians including 6 professional behaviors in the U.S.^4^ Research from the international perspective also recognized professionalism as a multi-dimensional construct embedded in social-cultural contexts.^5^ Academic Medicine published two volumes of the collection of 50+ empirical studies and perspectives on medical professionalism in 2011 and 2017.^1^^, ^^2^ While the 2011 volume reflects the shift from the focus of the professional as a collective entity to professionalism definitions at the individual, contextualized levels,^6^^, ^^7^ the 2017 volume expanded these perspectives by incorporating racial, cultural, and multinational perspectives and highlighting the importance of professional identity formation.^8^^, ^^9^

Empirical studies on professionalism in medical schools focus on diverse aspects and have identified various challenges, such as difficulties in teaching and evaluating professionalism, differing student and faculty perceptions of professionalism, and peer-assessment of professionalism in clerkships.^10^^, ^^11^^, ^^12^ Some of the studies focused on professionalism in small groups. For example, Emke et al. examined the relationships between self- and peer assessment of professionalism in Team-Based Learning (TBL) activities and found that standalone and simultaneous peer and self-assessments were highly correlated.^13^ However, a small group of individuals consistently rated themselves higher than their peers rated them. Curran and his collaborators examined the internal structure of a peer assessment tool regarding professionalism and investigated the overall satisfaction with peer assessment.^14^ Their study found mixed feelings about peer assessment and identified a lack of constructive feedback as a limitation. A systematic review was conducted to examine the utilization of small-group peer feedback in student learning and professional development.^15^ The review concluded that peer feedback was a reliable assessment for professionalism and aided in the development of professional behavior.

Professional attributes and behaviors are highly dependent on interpersonal interactions. Small-group learning modalities, such as TBL, in which students work together in teams to apply knowledge toward solving relevant problems, and case-based learning (CBL), in which students work together to explore and discuss a clinical case, rely on the collaborative efforts of group members to promote learning.^16^ In CBL and TBL, students have frequent, close contact with peers, which lends valuable and important opportunities to observe peers’ behaviors and attitudes. Due to the feature of intimate, close relationships in the small group, CBL and TBL provide unique opportunities for students to observe and reflect on their peers’ professionalism and provide feedback for each other to grow and learn. The pandemic forced many medical schools to adopt virtual modes for conducting TBL and CBL. It is not clear whether students demonstrate professional behaviors differently in an online setting due to the lack of a physical presence. Student reflections and feedback provide unique insights into peers’ behaviors. Although studies on professionalism have been conducted, few have directly compared professionalism in the face-to-face and virtual settings and examined how online learning impacts students’ perceptions and behaviors related to professionalism. The aim of this study is to determine whether and how preclinical students’ perceptions and behaviors related to professionalism in small group activities, including CBL and TBL, shifted as a result of the shift from face-to-face to virtual learning as a result of the COVID-19 pandemic.

## Methods

This study was determined to be exempt from institutional review board (IRB) review by CMU’s IRB. The IRB approval exemption from the university was received prior to data collection.

As synthesized earlier, the literature highlights contextualized, social-cultural perspectives on professionalism. As such, in the following, we start with an elaboration of the specific educational context where the study was conducted. We then explain two separate stages of the mixed-methods explanatory design, with a survey followed by focus groups. In an explanatory design, quantitative data is first collected and analyzed, followed by qualitative data collection and analysis. The qualitative stage, which builds on the quantitative results, helps to explain and elaborate on the quantitative results in more depth.^17^ The results of two stages are merged in the discussion. Our design gave more weight to the qualitative stage from the focus groups in order to explain the initial quantitative results and expand our understanding of the phenomenon in more depth. Due to the sequential nature of the study, we first describe data collection and analysis for Stage one followed by the methods used for Stage two in the following.

### Educational context

Central Michigan University’s College of Medicine (CMED), which is located in the Midwest of the United State, adopts an integrated curriclum with a series of interdisciplinary, systems-based course blocks. Each course block incorporates a variety of teaching methods, and CBL and TBL comprise nearly 50% of contact hours on average. At CMED, professionalism is defined as “a commitment to carrying out professional responsibilities, adherence to ethical principles, and sensitivity to all individuals.” Seven educational program objectives address this competency, including demonstrating respect, accountability, sensitivity, enhancing team functioning, giving and receiving feedback, respecting patients’ privacy, and demonstrating a commitment to ethical principles. CMED policies and the student handbook define professionalism and provides examples of professional behaviors such as “handing in assignments on time” and “developing successful working relationships with preceptors, staff, and peers by accepting constructive feedback.” In the pre-clinical phase, students receive teaching and guidance on maintaining their professional conduct through course sessions, orientation sessions, and learning modules. This content addresses expectations related to professional dress code, attitude, and behaviors when the students work with their peers in small groups.

In this current study, we compared student perceptions of professionalism in two 6-week courses, Reproductive System and Human Development (delivered face-to-face, January to February, 2020, pre-COVID-19) and Renal and Endocrine Systems (delivered online, April to June, 2020, during COVID-19). Students were in the same group for each course. At CMED, individual students prepare for TBL by reading pre-assigned material before participating in individual- and group-based TBL activities each week. After that, the same group participates in CBL by collectively working through patient cases. Finally, each group works on additional TBL sessions based on the cases.

### Data collection and analysis at the quantitative stage

Stage One used a retrospective approach based on the data that were obtained from 13 small groups of 8 or 9 students who participated in CBL and TBL exercises in these two 6-week courses in early 2020. At the end of each course, students completed peer evaluation surveys assessing their peers’ professionalism behaviors using a 5-point Likert scale ranging from “never”, “rarely”, “sometimes”, “often”, to “always.” The survey contained six items related to professional behaviors, including showing punctuality and remaining with the team, giving feedback, accepting feedback, showing respect and sensitivity, demonstrating integrity and accountability, and interacting professionally. Completion of peer evaluations was required, and all students (n = 101), 51 female and 50 male, completed the survey. Responses were analyzed using SPSS 26. Due to the ordinal data and violation of normality of the responses, the nonparametric Wilcoxson signed-rank test was used to compare students’ responses in the two settings.

### Data collection and analysis at the qualitative stage

After the quantitative data were collected and analyzed completely, the research team reviewed the results of the quantitative data, discussed the findings with statistical significance, and developed a semi-structured interview protocol. The interview protocol essentially asked about three major areas: perceptions regarding results from the quantitative findings, overall perceptions of how the change from face-to-face to online learning impacted professionalism, and perceptions of whether and how their determination of the degree of survey responses evolved in the online setting. The semi-structured interviews provided the opportunity to probe for clarification and explore participants’ perceptions in depth.

A purposeful sampling approach was used to recruit participants considering gender and test performance. Six groups with 27 volunteers in total were recruited in late 2020. Focus groups were conducted virtually, ranging from 45-60 minutes. Among the 27 participants, there were 11 males and 16 females (see Table 1). Students’ average course performance ranged widely from 70% to 92%. The interviews were transcribed verbatim and analyzed using a thematic inductive coding method.^18^ To develop a coherent synthesis of the data corpus, we first coded and recoded to discover accurate words or phrases to represent the data using the initial coding. In the initial coding, we broke down qualitative data into discrete parts, closely examined them, and compared them for similarities and differences. Then, we used axial coding to describe a category’s properties and dimensions and explore how the categories relate to each other.^19^ After a number of close readings recursively, some codes were merged because they were conceptually related and infrequent codes were dropped because they were deemed marginal.^20^ Categories were then derived directly and inductively from the raw data. Representative direct quotes are reported in the results below with the corresponding student number in Table 1.

**Table 1:** Composition of the six focus groups by number of students and gender

Focus Group	Student	Gender	Focus Group	Student	Gender
FG1	S1	Female	FG4	S15	Male
FG1	S2	Male	FG4	S16	Female
FG1	S3	Female	FG4	S17	Male
FG1	S4	Female	FG4	S18	Female
FG1	S5	Male	FG4	S19	Female
FG2	S6	Female	FG5	S20	Male
FG2	S7	Male	FG5	S21	Male
FG2	S8	Male	FG5	S22	Female
FG2	S9	Female	FG6	S23	Female
FG2	S10	Female	FG6	S24	Female
FG3	S11	Female	FG6	S25	Male
FG3	S12	Male	FG6	S26	Female
FG3	S13	Female	FG6	S27	Female
FG3	S14	Male			

## Results

### Quantitative Stage

Cronbach’s alpha internal consistency reliability coefficient with the six 5-point Likert-scale items was calculated. Results showed sufficient reliability with two courses (α = .891 and .911 respectively), indicating that the six items broadly reflected the dimension of professionalism. Average peer-evaluation responses were calculated for each student, which was then compared in the two settings. Overall, students’ assessment of their peers was positive. The mean of the responses ranged from a low of 4.38 to a high of 4.85. Results showed a significant difference between the two settings for only one question, “Arriving on time and remaining with team during activities” (Z = -6.211, n = 101, p < .001), which decreased from 4.74 in the face-to-face setting to 4.38 in the online setting.

**Table 2:** Wilcoxson signed-rank test

Survey Question	N	Face-to-face		Online		Z	p- value
		Mean	SD	Mean	SD		
Arriving on time and remaining with team	101	4.74	.33	4.38	.55	-6.211	.000
Giving constructive feedback	101	4.72	.32	4.73	.29	-.618	.536
Accepting constructive feedback	101	4.72	.40	4.75	.27	-.700	.484
Showing respect and sensitivity to others	101	4.81	.25	4.83	.30	-1.137	.256
Demonstrating integrity and accountability	101	4.85	.17	4.84	.27	-.402	.688
Interacting professionally with team members	101	4.81	.22	4.84	.20	-1.584	.113

### Qualitative Stage

Using the standard thematic inducive coding process, five major categories emerged from the qualitative data: 1) punctuality and participation, 2) camera usage, 3) dress code and conversational styles, 4) multitasking, 5) engagement and accountability.

### Punctuality and participation

The statistical difference was further examined by exploring participants’ perceptions in depth. Generally speaking, the focus groups confirmed the finding of reduced punctuality and remaining with the team from the Likert-scale responses. Participants agreed that their peers showed less punctuality and attendance as stated, “professionalism definitely probably decreased. It’s just easier for people to show up late or not show up at all” (FG3, S11) and “I know at least this year, we've, in my group, had a couple of students sleep through case” (FG1, S2). As one student elaborated,

“I do find people are tardier now getting on onto cases, whereas when we were in person, you knew you had to be there at 8 am, and you knew how long the drive took, and you were there for the most part at 8 am. Now, I have found that…people kind of will trickle into case and we'll start a few minutes late.” (FG1, S3)

Furthermore, participants explained their changed definitions and lowered expectations about punctuality and attendance (e.g., “[people] are more lenient” FG3, S11 & S14;

“it feels a lot, less like, rude to not show up to a meeting online” FG5, S20) compared with face-to-face CBL and TBL. It was also more difficult for students to assess their peers’ participation. One student explained the difficulty in interpreting a lack of participation by a peer: “you know someone is doing something else and that you don't necessarily know if it's because they don't understand what's going on and they're trying to look more into it or if they're just not there” (FG2, S10).

**Camera usage**

In addition to punctuation and participation, camera usage emerged as a distinct theme from the interview data. At CMED, students were not required to use their cameras during small-group sessions. The participants in focus groups discussed how the online setting without cameras constrained communications and collaborations and lack of facial expressions made difficult to see “someone's reaction if they got a question wrong or if they are trying to explain something” (FG1, S2). As one participant explained, “it becomes a little bit harder to gauge whether or not people are actually participating or if they're engaging in active listening or…what's going on behind the screen when they don't have their cameras on” (FG2, S7).

The participants discussed two different behavioral patterns and how these patterns impacted their perceptions of professionalism. First, students who turned off their camera but contributed to group activities were considered professional, as stated, “I think is the fact that we…don't turn our cameras on, I mean, still it’s fine. Like, I don't mind if your camera's off and you're still contributing, or you want to talk if you have questions” (FG5, S22). Second, those students who turned off their camera and did not contribute were considered unprofessional. The participants expressed that they understood there were occasions which required camera off or being muted such as “a lawnmower going in the background, or like a dog barking for 10 minutes straight” (FG4, S19); however, the participants believed being muted with camera off without active engagement was not professional:

“There’re just people in my group who, no camera, will not say anything, will not write anything on a document for days in a row……So, by not showing up, not asking questions, not answering questions, you're doing a disservice to the rest of your group. And I feel like there's just like, no good way to do it in online, because how do you know if somebody's there and just not saying anything or if they turn the camera [off] and walk away from it and are just spinning their wheels?” (FG5, S22)

**Dress code & conversational styles**

The participants were fully aware how the online setting changed the way they dressed for classes and how they talked with each other. Although the school has a dress code, students stated, for example, “I don't necessarily dress up every day for class, as opposed to if we had to go in person” (FG2, S9). Their judgment about professionalism became more contextualized, less joking, and less judgmental. For example, the following student explained while he believed professionalism was deteriorating, a new dress code of wearing pyjamas was acceptable.

“I don't think anybody expects anybody to wear anything more than a T-shirt, but, I mean, we were wearing jeans and T-shirts in school. So, I don't think if you’re, I mean, if you're

wearing pyjamas and you're at home, as long as you're not like, in your underwear, as long as you're wearing, you know, whatever. I feel like that's kind of the new professional as long as you're like, you know, clothed.” (FG5, S22)

Some students also expressed concerns about how their words or actions could be interpreted by their peers with fewer visual cues. As such, they were careful with their tones and word choices in group discussions.

“So, we were very hesitant to say certain things in a joking manner because no one has their cameras on, so we don't know if someone's actually going to take like, a joke seriously. And maybe it could be interpreted, like, in a wrong way, and we're not able to gauge their reactions. So, at least for me, I feel like I toned down how I speak to others, to become more professional.” (FG1, S2)

**Multitasking**

Multitasking emerged as a theme with controversial perceptions among the focus group participants. The students talked about doing other things such as “do some [weight] lifting” (FG5, S21) or “doing a load of laundry” (FG3, S13). Some participants found multitasking to be convenient and appropriate, for example, “there is a little bit of multitasking, but usually people prioritize the case, so I think it's it still works out” (FG2, S9). Others deemed such behaviors as unprofessional and tried to get rid of distractions, as one student described, “I have to literally put my phone in a different room to keep myself from just looking at it and accidentally losing part of the time, when I'm supposed to be paying attention to someone answering a question” (FG6, S24). For example, the following two participants showed opposite attitudes toward multitasking.

“Professionalism is decreased, but not for, like, respect or kindness or anything like that. But just that, you can take a call. I had a dentist appointment, and I took case on a hot spot on my phone in my computer in my car. Like, I was in a parking lot doing class, and it worked fine. And I know a lot of people do that, you know, different places or hopping around or things like that.” (FG3, S13)

“I definitely think professionalism changed and, like, I know I've even found myself guilty of this now that we're online and, like, we don't normally turn our cameras on as a group. Like, I find myself doing other things that may not be focused on class or like, I'll be like, making lunch instead of, like, listening in TBL, which, is not great.” (FG26, S23)

### Engagement & accountability

The virtual setting impacted how students perceived their ability to provide meaningful feedback and demonstrate accountability. The participants expressed that they were not sure when to chime in, how to chime in, and whether they should give peers the benefit of the doubt due to the use of the online setting. Compared with the face-to-face setting, “you aren’t, as, like, accountable, and it's easier for them [peers] just to not participate at all. So, if they just want to shut off their camera and, like, not talk the whole case, you can't really do much about it. But, like, in person, people were more willing to at least somewhat talk” (FG3, S11). The following student elaborated on the difficulty to be engaged and provide feedback:

“So, it's kind of hard to just say what you're thinking, because then you don't know if someone's going to talk and then you kind of have to be hesitant on when you want to talk versus letting someone else talk. So, I find myself, kind of being hesitant and saying what I want to say if I was in person, because there's not that lag time or, you know, the body language and stuff like that. So that's kind of where I think sharing information kind of goes down and sharing your own opinion and stuff.” (FG2, S9)

**Discussion**

The mixed-methods design adopted in this study is helpful to explain quantitative results and expand understanding and interpretations of professionalism in more detail. The study used retrospective peer evaluation results and then moved to constructivist assumption to investigate multiple perspectives and in-depth exploration. To recap, the quantitative results showed significant differences regarding arriving on time and remaining with team between face-to-face and online settings, despite lowered expectations for the online setting as described by focus group participants. Participants observed a general decrease regarding punctuality and attendance in the online setting. Furthermore, the qualitative interview results indicated how the definition of professionalism became contextualized, significantly influenced by the virtual learning environment. Participants recognized the blurred boundary between professional and personal spaces in the online setting. Some participants viewed multitasking as convenient and appropriate while others deemed such behaviors as unprofessional. Overall, even though the CMED student handbook delineates regulations and requirements for professional conduct and presentation, details and nuances that were perceived and considered by the participants go beyond these regulations.

In summary, the results showed that the shift to the online setting significantly impacted students’ perceptions and behaviors toward professional conduct in the process of establishing and maintaining their professional relationships with their small group members. This is consistent with the findings from the two *Academic Medicine* volumes that large-scale technological, social, and political changes might be having substantial effects on medicine’s status as a profession and on the identity of physicians as professionals.^1^^, ^^2^ Professionalism in the online setting became more intangible and blurred. This is partially due to the format of the online setting, which does not distinguish the personal space from the professional space; hence, it becomes more challenging to define professional behaviors and keep the boundaries between personal and professional spaces. Such results are consistent with the literature that professionalism is contextualized and existing definitions lack in focus or details.^21^^, ^^22^ The continually shifting nature of the organizational, social-cultural, and technological milieu make it challenging to hold these definitions as definitive.^2^

Results from this study also showed the dynamic process of professional identity formation, although at the early stage of medical students’ career path. Professional identity formation has received considerable attention in recent years. Irby and Stanley defined professional identify formation as “an adaptive, developmental process occurring at individual (psychological) and collective (sociological) levels that socialize learners into thinking, feeling, and acting like a physician.”^23^ In this study, the participants expressed sensitivity and being less judgmental when giving formative feedback, indicating the emergence of their adaptive thinking process. The change from in-person to online clearly effected the way how students assessed their peers’ professionalism. They were aware of the impact of the fundamental technological change and tried to justify such changes.

Professional identity formation for each individual student is not static, but rather a fluid process requiring flexibility and resilience. Navigations and negotiations of their personal and their peers’ behaviors in the online learning setting during the pandemic provide an opportunity for students to reflect on new values and identities, and create compromise between the two, as argued by Larry May in terms of “legitimate compromise.”^24^He criticized professional codes for seeking to impose regulations that do not consider various conditions of particular conflicts and advocated a cooperative and conciliatory approach to inquire into professionalism.

Professional identity formation is a process; it entails intentional discussions collectively at the college level and should include faculty and administrators who teach professionalism and serve as role models. Professional identity formation may also be inspired by gaining different perspectives from various stakeholder groups beyond the local community. Ultimately, intentional communications about professional conduct at the collective level with the context are vital for individual professional identity formation, which is dependent on interpersonal interactions and heavily embedded in social-cultural contexts.

### Limitations

This study has several limitations. First, the quantitative stage was based on the retrospective peer assessment data, which provided limited information even though the study found appropriate reliability evidence. This was a small-scale study focusing on only one educational context with its own characteristics regarding student population, learning approaches, and teaching features. Professional attitudes and behaviors may vary when using different populations in different educational contexts with different policies and practices.

## Conclusions

The competencies to engage in collaboration and function effectively in a professional manner are essential attributes of any good physician. Medical education does not just entail the delivery of medical knowledge and skills but also includes the cultivation of professional attitudes and behaviors as students develop their professional identities as physicians. Given the context that the pandemic forced medical schools including CMED to adopt virtual CBL/TBL, it is vital to examine how the shift from face to face to online learning affected students’ perceptions of professionalism and professional behaviors in small groups.

Unprofessional attitudes and behaviors negatively impact learning process, performance, teaching, and the larger learning environment. This study provides empirical evidence confirming that the shift to the online setting significantly influenced students’ professional behaviors in the process of establishing and maintaining their professional relationships with their small group members, partially due to the unclear guidelines of professionalism in the local context. Further research will be needed as the pandemic continues to evolve to see how the change among virtual, face-to-face, and blended models of medical education affects students’ attitudes and behaviors in professionalism. It is also important to examine whether this almost fully virtual-learning class demonstrates professionalism when they rotate and practice in clinical settings as Year 3-4 students and residents.

This paper provides important implications at CMED and beyond. First, the timely investigation of professionalism in these two settings has accumulated information about how online setting influenced students’ perceptions and behaviors regarding professionalism. It confirms that the definition of professionalism is contextualized and situated. Expectations or additional specifics under the online circumstances, hence, need to be clarified and reinforced in school policies and curriculum (e.g., punctuality, dress code, and accountability). Second, the results provide directions about the importance of fostering professional identify formation and discussing some of the controversial issues (e.g., the use of camera, multitasking) related to professionalism in the local context, with or without the impact of the COVID-19. It is essential for CMED and other medical schools to reflect on the feature of its student population and organizational culture to discuss perceptions and conflicts regarding professionalism. Professionalism and professional identify formation must be aligned with school missions and cultivated by both who deliver the curriculum and who receive the training. Without inclusive and transparent conversations during the journey both curriculum and assessment of professionalism will come under challenge. It must be noted that this was a small-scale study focusing on only one educational context with its own characteristics regarding student population, group dynamics, and teaching features. This group of students had limited experiences with online learning. Perceptions may vary when using different samples in different educational contexts at different counties.

### Acknowledgement

We are grateful for Dr. Mariana Rosca who helped with brainstorming and data collection as well as CMED students for sharing their experiences. We thank the editor and reviewers for their constructive comments.

### Conflict of Interest

The authors declare that they have no conflict of interest.
